# Application of an Intraoperative Limb Positioner for Adjustable Traction in Both-Column Fractures of the Acetabulum: A Technical Note with Clinical Outcome

**DOI:** 10.3390/jcm12041682

**Published:** 2023-02-20

**Authors:** Joon-Woo Kim, Chang-Wug Oh, Kyeong-Hyeon Park, Won-Ki Hong, Sung-Hyuk Yoon, Gwang-Sub Lee, Jong-Keon Oh

**Affiliations:** 1 Department of Orthopedic Surgery, School of Medicine, Kyungpook National University, Kyungpook National University Hospital, Jung-gu, Daegu 41944, Republic of Korea; 2 Department of Orthopedic Surgery, School of Medicine, Korea University Guro Hospital, Seoul 10408, Republic of Korea

**Keywords:** acetabular fracture, both-column fracture, intraoperative traction, limb positioner

## Abstract

Traction of the ipsilateral leg is usually required to facilitate fracture reduction while operating both-column acetabular fractures. However, it is challenging to maintain constant traction manually during the operation. Herein, we surgically treated such injuries while maintaining traction using an intraoperative limb positioner and investigated the outcomes. This study included 19 patients with both-column acetabular fractures. Surgery was performed after the patient’s condition had stabilized, at an average of 10.4 days after injury. The Steinmann pin was transfixed to the distal femur and connected to a traction stirrup; subsequently, the construct was affixed to the limb positioner. A manual traction force was applied through the stirrup and maintained with the limb positioner. Using a modified Stoppa approach combined with the lateral window of the ilioinguinal approach, the fracture was reduced, and plates were applied. Primary union was achieved in all cases at an average of 17.3 weeks. The quality of reduction at the final follow-up was found to be excellent, good, and poor in 10, 8, and 1 patients, respectively. The average Merle d’Aubigné score at the final follow-up was 16.6. Surgical treatment of both-column acetabular fracture using intraoperative traction with a limb positioner yields satisfactory radiological and clinical outcomes.

## 1. Introduction

Both-column fracture of the acetabulum is relatively common among acetabular fractures and is mainly caused by high-energy trauma [1,2]. During open reduction and internal fixation, intraoperative traction of the ipsilateral leg is required in most cases to facilitate fracture reduction and stabilization. In particular, in acetabular fractures associated with central dislocation of the femoral head, adequate reduction in the fracture is challenging without traction. Methods for applying intraoperative traction in pelvic and acetabular surgery are as follows: taking help from a skilled assistant for manual traction, using a universal distractor or external fixator, and using an on-table frame [3,4,5,6]; however, there is no consensus on an ideal technique [7]. In general, intraoperative traction is entirely dependent on surgical assistants because it is the simplest and most easily reproducible method, requiring no special equipment. However, it is not easy for humans to perform precise and continuous traction with constant force throughout the operation. Moreover, the surgical assistant can easily be exhausted, and the need for extra operating room personnel is another drawback.

Limb positioners, which were originally designed for upper extremity surgery and arthroscopic procedures, have been increasingly used in lower extremity procedures because they can be easily adjusted intraoperatively [8]. We hypothesized that both-column fractures of the acetabulum can also be effectively managed with intraoperative traction using a limb positioner. Accordingly, we describe our technique, which—to the best of our knowledge—has not yet been reported in patients with both-column fractures of the acetabulum. This study aimed to report on the novel use of a limb positioner as an intraoperative reduction aid for both-column fractures of the acetabulum as well as its clinical outcomes.

## 2. Materials and Methods

Our study included 19 patients, including 10 men and 9 women (mean age, 54 [range, 21–89] years) with both-column fractures of the acetabulum. Notably, the causes of the injury were traffic accidents and fall from a height in 13 and 6 patients, respectively. Of the 19 patients, 11 (57.8%) had a central dislocation of the femoral head. The average follow-up period was 30.5 (range, 12–60) months (Table 1). All surgeries were performed by two experienced surgeons (J.-W.K. and C.-W.O.), and the surgical team usually consisted of one surgeon and two assistants, sometimes one surgeon and one assistant.

### 2.1. Operative Technique

1. Preparation

**Figure 1:**
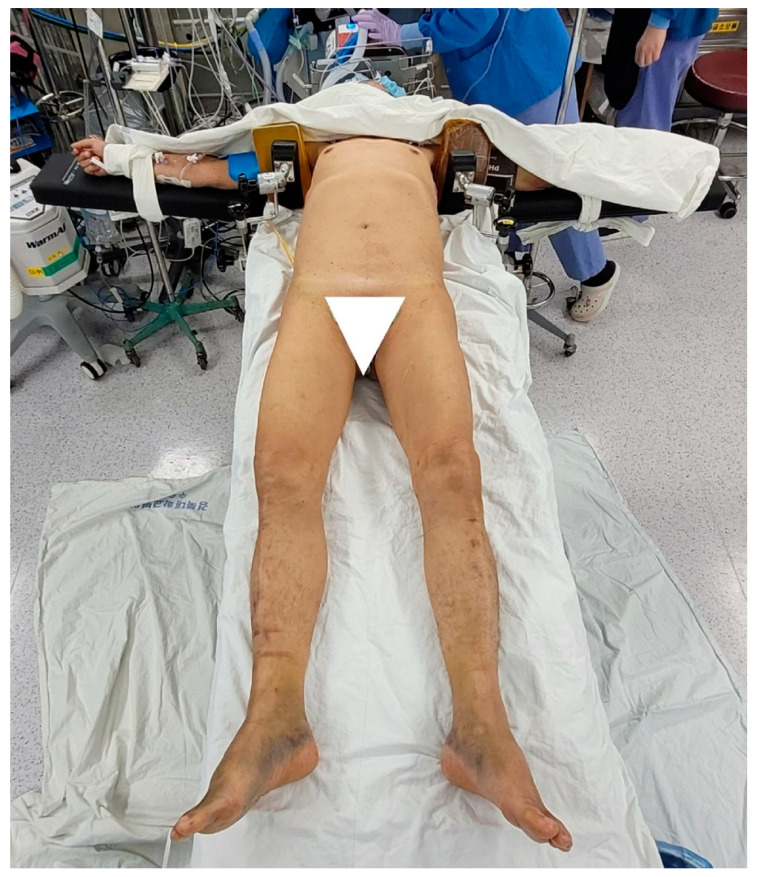
After anesthesia, the patient was placed in a supine position with both arms abducted at 90°. A shoulder support was placed on both axillae with a jelly pad to prevent the patient from getting pulled down while maintaining longitudinal traction with a limb positioner.

After the induction of general anesthesia, the patient was placed in a supine position on a radiolucent table. The patient’s both arms were placed on the arm board at 90° abduction. A shoulder support was placed on both axillae with a jelly pad to prevent the patient from getting pulled down while maintaining longitudinal traction with a limb positioner (Figure 1). Further, an image intensifier was introduced from the contralateral side.

The entire ipsilateral lower limb was then prepared and freely draped to facilitate the intraoperative reduction maneuver. Notably, sterile draping was extended proximally to the subcostal region. A pillow was placed underneath the popliteal fossa for slight flexion of the hip in order to relax the iliopsoas muscle.

2. Traction with a limb positioner

To insert a pin for traction, the knee was flexed to 30° with neutral rotation. Using a pointed scalpel, a stab incision was made through the skin on the medial side 2–3 finger breadths above the superior pole of the patella. After placing a 3.2 mm Steinmann pin on the drill, insertion was made parallel to the joint line from the medial to lateral sides. Further, after driving the Steinmann pin through the bone and ensuring that the pin had penetrated the far cortex, another stab incision was made on the overlying skin, coinciding with the expected exit of the pin. After the Steinmann pin was completely out, the tension on the skin at the entry and exit points was checked. A small relieving incision was additionally performed in case of excessive tension.

**Figure 2:**
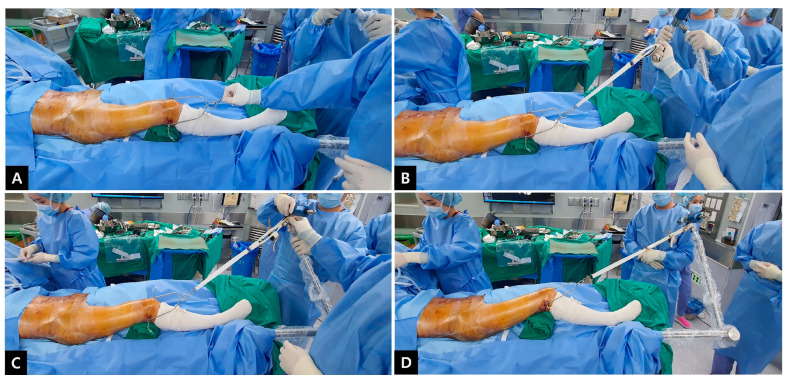
After transfixing the Steinmann pin in the distal femur, it was connected with a traction stirrup (**A**). The Stein-mann pin-traction stirrup construct was affixed to the limb positioner (**B**). Sufficient manual traction force was applied through the stirrup (**C**). The limb positioner was locked while maintaining traction (**D**).

**Figure 3:**
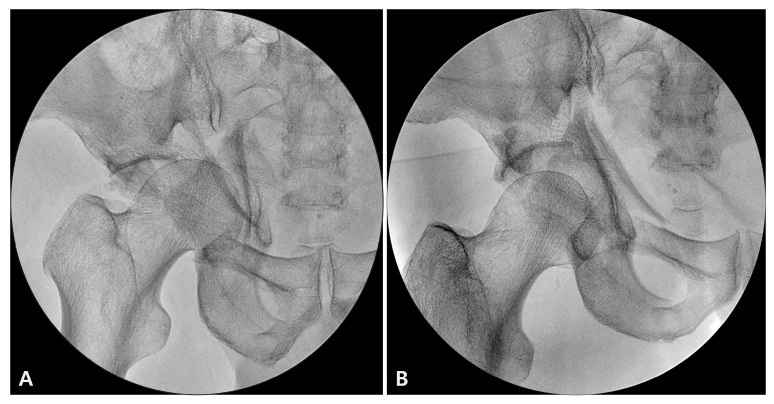
Intraoperative image before (**A**) and after (**B**) traction.

The Steinmann pin was transfixed and then connected with a traction stirrup and affixed to the limb positioner (The Spider Limb Positioner, Smith and Nephew^®^, Andover, MA, USA). Sterility was assured by first covering the limb positioner with the manufacturer’s sterile drape and then proceeding with standard sterile pelvic draping (Figure 2). Subsequently, sufficient manual traction force was applied through the stirrup, and the degree of reduction was confirmed using an image intensifier (Figure 3). The stirrup was then connected to the pneumatic limb positioner and locked while maintaining traction (Figure 2D and Video S1).

3. Reduction and fixation

We used a modified Stoppa approach combined with a lateral window of the ilioinguinal approach. First, we aimed to reduce the displaced anterior column to the posterior ilium. A 5.0 mm Schanz screw was inserted in the anterior inferior iliac spine, and the iliac wing was internally rotated. The elevated anterior column fragment was squeezed out using a ball spike pusher. A 5–6-hole reconstruction plate or small locking compression plate was undercontoured and placed at the junction of the fracture line along the pelvic brim. The distal part of the plate was placed on the free anterior column fragment, and cortical screws were fixed to the proximal portion of the plate—the stable portion of the posterior ilium. With the tightening of the screws, the under-bent plate pressed the anterior column fragment into alignment with the intact ilium. Cortical screws were then fixed into the distal portion of the plate while exercising caution to avoid pulling the anterior column fragment. We also performed reduction and fixation of the iliac wing with a lag screw or reconstruction plate, if required.

Subsequently, the posterior column was reduced. Notably, as this column was already almost reduced by ligamentotaxis via traction through the limb positioner in most cases, only fine adjustment or augmentation was required. The pelvic arm of the collinear reduction clamp was placed in the lesser sciatic notch from the lateral window of the ilioinguinal approach. Further, the collinear reduction clamp was assembled with the pelvic arm and gently squeezed while observing the reduction status via the Stoppa window. After confirming that the quadrilateral surface was adequately reduced to the anterior column via direct visualization, a 3.5 mm long lag screw was placed in the direction of the ischial spine. We made it a rule to place at least two screws for the posterior column fixation.

Finally, a curved 12-hole pelvic reconstruction plate was contoured and applied along the pelvic brim, from the innominate bone adjacent to the sacroiliac joint to the pubic tubercle. Remarkably, the plate was introduced from the lateral window of the ilioinguinal approach in the direction of the distal Stoppa incision. The cranial- and caudal-most screws were placed to buttress and stabilize the reduced anterior column fragment. An additional posterior column screw was placed through the plate hole or separately next to the plate hole if required.

The fixation status was confirmed using an intraoperative image intensifier in the anteroposterior, iliac wing, and obturator oblique views. If a large posterior wall fragment was present or the posterior column reduction was unsatisfactory, they were corrected and stabilized using a separate posterior approach. After completion of all fixations, the traction was released and a final radiographic assessment was performed before wound closure.

### 2.2. Postoperative Management and Assessment

Patients were encouraged to sit up within the first 24–48 h after surgery, and active hip and knee joint motions were advised. Partial weight bearing was allowed with crutches for 8 weeks after the operation, and this progressively increased to full weight bearing after 8 weeks. Further, sequential follow-up radiographs of the anteroposterior, iliac wing, and obturator oblique views of the pelvis were obtained at regular intervals of 4–8 weeks.

In radiological evaluations, healing rate, time to union, quality of reduction, and complications were assessed. Based on these findings, the quality of reduction was graded as excellent, good, fair, and poor according to Matta’s criteria [9]. Moreover, the clinical results were graded as excellent, good, fair, and poor according to the modified Merle d’Aubigné scoring system (excellent, 18; good, 15–17; fair, 12–14; poor, <12), which is based on the assessments of pain, walking, and range of motion.

## 3. Results

Operative fixation was performed at an average of 10.4 (range, 4–22) days after patients were appropriately resuscitated and optimized for surgery. Seven patients had multiple fractures, including spine, forearm, tibial, and ankle fractures. Two patients sustained various chest traumas, such as flail chest, pneumo-/hemothorax, and multiple rib fractures. Two patients sustained a liver injury that required emergency intervention.

Overall, 16 of the 19 patients underwent surgery via the anterior approach alone, whereas three patients required additional posterior fixation through a separate posterior approach. The mean operation time was 208.6 min (range, 150–290). Primary bone union was achieved in all cases at an average of 17.3 (range, 15–20) weeks. The quality of reduction assessed by Matta’s criteria at the final follow-up was excellent, good, and poor in 10, 8, and 1 patients, respectively. Notably, all patients achieved excellent or good functional outcomes with a median Merle d’Aubigné score of 16.6 (range, 15–18), except for two patients.

These two patients (10.5%) underwent hip arthroplasty at 5 and 11 months postoperatively, respectively. One patient had a severe femoral head impaction at the time of injury, and osteonecrosis of the femoral head, followed by secondary arthritis, was found to be rapidly progressing. The other patient sustained severe comminution of the acetabular cartilage. Although the postoperative reduction status was relatively satisfactory, joint space narrowing gradually progressed and osteoarthritis eventually developed with complaints of severe pain.

Complications caused by continuous traction, such as nerve or vascular damage, pin site problems, or pressure sore, were not observed in any case.

## 4. Discussion

Reduction is the first and most important step in acetabular surgery. Intraoperative traction is essential to neutralize the deforming force that caused the fracture, and it facilitates the reduction. Various intraoperative traction methods have been described in the relevant literature, including the use of a surgical assistant to provide intermittent manual traction, an external fixator, a fracture table, or an on-table frame [7]. A skilled assistant can apply manual traction, but the assistant can easily be exhausted, and the need for additional operating room personnel is another drawback. Moreover, a previous study reported that the major disadvantage of using a radiolucent table is the need for manual traction; thus, it requires a minimum of two or three assistants [10]. In addition, it is difficult for a human to apply a constant force throughout the operation. In contrast, the benefit of using the fracture table is that constant and precise traction can be maintained indefinitely, although an additional surgical assistant is still required to operate the table. However, the design of the fracture table limits certain movements of the extremity and interferes with certain fluoroscopic views [11,12,13]. Notably, an on-table frame can be used for this purpose, but force vectors are two-dimensional [5]. Moreover, external fixators or distractors can be used, although traction is most commonly provided along a single defined vector in these techniques [3,4,5,6]. In contrast, the method described in our study does not require additional personnel, and the number of assistants can be decreased. Before using limb positioner traction, our surgical team for a pelvic-acetabular fracture usually consisted of one surgeon and three assistants, whereas two assistants are sufficient after using this method. In addition, it is easily adjustable and can be manipulated in multiple vectors simultaneously. An additional advantage is that the distraction direction, which facilitates fracture reduction, can be adjusted and maintained and, if necessary, easily changed during the operation. Similarly, compared with other table attachments that offer only leg movement, a particular advantage of the limb positioner is that the leg can be manipulated in rotation and flexion/extension while engaged [7]. In addition to having complete freedom of leg position when initially applying traction, it is easy to adjust it as often as preferred. Furthermore, since most centers performing limb surgery are generally furnished with a limb positioner, it is also considered to be cost effective to use this as a traction device in this respect.

The Spider Limb Positioner is a pneumatic arm with three fully articulated joints that uses compressed air or nitrogen to facilitate its static locking mechanisms. It was classically used for shoulder arthroscopic procedures. The foot pedal allows the surgeon to control the limb during surgery and is the means by which pressurized air or nitrogen is supplied to the pneumatic arm. Notably, the foot pedal unlocks the three joints simultaneously, allowing the repositioning of the limb in an infinite number of positions while maintaining a sterile field [8]. Additionally, the limb can be connected to and disconnected from the limb positioner while maintaining a sterile field throughout the procedure. Owing to the limb positioner’s unique ability to allow infinite positional adjustments in three dimensions, we aimed to use it for intraoperative traction. Furthermore, the limb positioner can support a maximum of 22.3 kg (50 lbs) [8], which is believed to provide sufficient strength for traction.

In a previous study of both-column acetabular fracture, the hip joint was congruent in 94.7% after surgery, which is comparable to our result [2]. However, they experienced 8.9% of iatrogenic nerve injuries and 60.7% of patients had the mean Merle d’Aubigné score of 15, and 25.8% of the patients diagnosed a joint failure, which is somewhat inferior to ours. The operation time in the current study was also relatively shorter than described in previous studies [2,14,15]. It is believed that this is because the operation can be performed while maintaining stable traction with the limb positioner, without repetitive actions for reduction.

To the best of our knowledge, no case series has described the use of a limb positioner as a reduction tool with clinical and radiological outcomes in both-column fractures of the acetabulum with adequate follow-up, although a previous case report described the technique of lateral traction for reduction of the medialized femoral head using a limb positioner [7]. Although intraoperative traction using a limb positioner may not have a significant effect on the clinical and radiological outcome, it is considered to be true that the surgical procedure can be convenient and efficient.

This study has some limitations. First, the study used a retrospective design and a small cohort size. Second, the unconventional use of a limb positioner for traction purposes is not authorized. However, considering that this is a novel attempt to introduce the limb positioner in acetabular fracture surgery, we believe that it deserves attention as it can provide acetabular surgeons with a new reliable traction technique. In addition, this can be a reasonable and safe alternative technique to maintain intraoperative traction when operating both-column fractures of the acetabulum. 

## 5. Conclusions

Surgical treatment of both-column fractures of the acetabulum using intraoperative adjustable traction with a limb positioner is considered an effective and safe method because it allows continuous traction with constant force throughout the surgery and without any traction-related complications. It also helps to reduce and stabilize the fracture, reduces the number of required operating room personnel, and yields favorable radiologic and functional outcomes.

## Figures and Tables

**Table 1:** Patient’s background and summarized results.

No.	Age	Sex	Injury Mechanism	Side	CDFH	Operation Time (Minutes)	Reduction Status (by Matta)	Union Time (Months)	Follow-Up Duration (Months)	Merle d’Aubigné Score	Complication
1	35	M	Driver accident	L	Yes	150	Excellent	16	40	15	
2	66	M	Pedestrian accident	R	Yes	240	Excellent	18	50	18	
3	89	F	Fall down	L	No	200	Good	17	30	18	
4	85	M	Motorcycle accident	R	Yes	180	Good	18	12	15	
5	60	F	Pedestrian accident	R	No	240	Excellent	16	32	15	
6	50	F	Pedestrian accident	R	No	190	Excellent	20	19	18	
7	55	M	Pedestrian accident	R	Yes	200	Good	20	60	15	
8	47	M	Pedestrian accident	L	Yes	250	Excellent	18	49	16	
9	21	F	Motorcycle accident	R	Yes	240	Excellent	15	29	15	
10	55	M	Fall down	L	No	180	Good	20	30	17	
11	58	M	Fall down	L	No	220	Excellent	18	24	16	
12	56	M	Pedestrian accident	R	Yes	240	Good	18	28	16	
13	71	F	Pedestrian accident	R	No	200	Good	16	23	15	ONFH
14	50	M	Fall down	R	Yes	155	Excellent	16	23	18	
15	25	F	Fall down	R	Yes	180	Excellent	18	30	18	
16	60	F	Driver accident	L	No	170	Good	18	26	18	
17	31	M	Fall down	R	Yes	240	Excellent	16	34	18	
18	64	F	Pedestrian accident	R	Yes	290	Poor	18	17	15	
19	44	F	Pedestrian accident	R	No	200	Good	13	24	18	Traumatic OA

M: male, F: female, CDFH: Central dislocation of the femoral head, ONFH: Osteonecrosis of the femoral head, OA: osteoarthritis.

## Data Availability

Source data may be shared upon reasonable request to the corresponding author.

## Supplementary Materials

The following supporting information can be downloaded at: https://www.mdpi.com/article/10.3390/jcm12041682/s1, Table S1: Patient’s background and summarized results; Video S1: The process of applying the limb positioner for intraoperative traction.
