# Supplementary material for: Application of an Intraoperative Limb Positioner for Adjustable Traction in Both-Column Fractures of the Acetabulum: A Technical Note with Clinical Outcome
Source: J Clin Med. 2023 Feb 20;12(4):1682. doi: 10.3390/jcm12041682 (PMC9965046; doi:10.3390/jcm12041682)
Supplement: Supplementary file 1 [file jcm-12-01682-s001.zip › Table S1.pdf]

Table S1. Patients' background and summarized results

No.	Age	Sex	Injury mechanism	Side	CDFH	Operation time (minutes)	Reduction status (by Matta)	Union time (months)	Follow-up duration (months)	Merle d'Aubigné score	Complication
1	35	M	Driver accident	L	Yes	150	Excellent	16	40	15	
2	66	M	Pedestrian accident	R	Yes	240	Excellent	18	50	18	
3	89	F	Fall down	L	No	200	Good	17	30	18	
4	85	M	Motorcycle accident	R	Yes	180	Good	18	12	15	
5	60	F	Pedestrian accident	R	No	240	Excellent	16	32	15	
6	50	F	Pedestrian accident	R	No	190	Excellent	20	19	18	
7	55	M	Pedestrian accident	R	Yes	200	Good	20	60	15	
8	47	M	Pedestrian accident	L	Yes	250	Excellent	18	49	16	
9	21	F	Motorcycle accident	R	Yes	240	Excellent	15	29	15	
10	55	M	Fall down	L	No	180	Good	20	30	17	
11	58	M	Fall down	L	No	220	Excellent	18	24	16	
12	56	M	Pedestrian accident	R	Yes	240	Good	18	28	16	
13	71	F	Pedestrian accident	R	No	200	Good	16	23	15	ONFH
14	50	M	Fall down	R	Yes	155	Excellent	16	23	18	
15	25	F	Fall down	R	Yes	180	Excellent	18	30	18	
16	60	F	Driver accident	L	No	170	Good	18	26	18	
17	31	M	Fall down	R	Yes	240	Excellent	16	34	18	
18	64	F	Pedestrian accident	R	Yes	290	Poor	18	17	15	
19	44	F	Pedestrian accident	R	No	200	Good	13	24	18	Traumatic OA
