# Supplementary figures and images for: Application of an Intraoperative Limb Positioner for Adjustable Traction in Both-Column Fractures of the Acetabulum: A Technical Note with Clinical Outcome
Source: J Clin Med. 2023 Feb 20;12(4):1682. doi: 10.3390/jcm12041682 (PMC9965046; doi:10.3390/jcm12041682)

## Slide 1
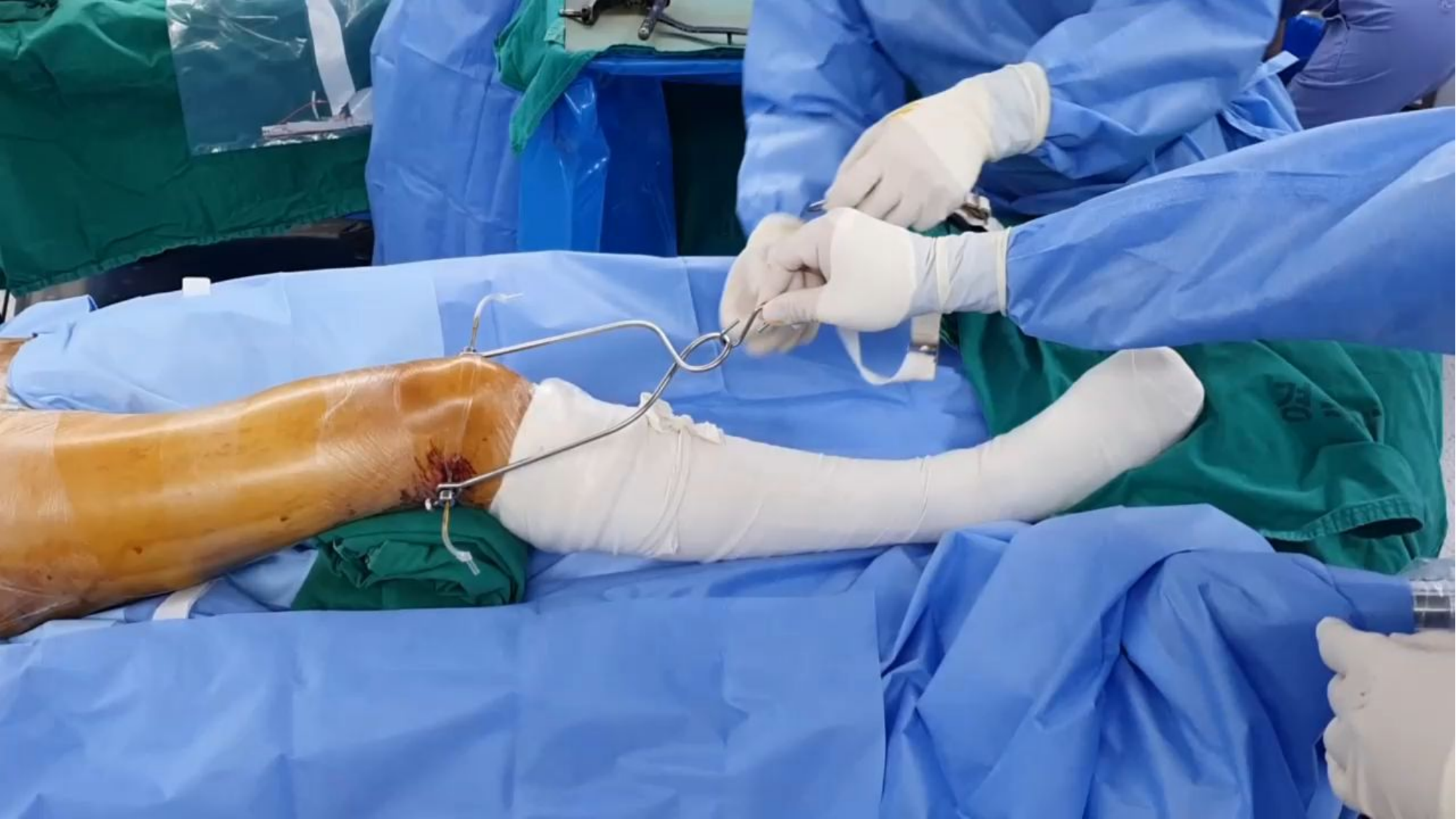

Supplement: Supplementary file 1 [file jcm-12-01682-s001.zip › Video 1.pptx]
